# ECRG4 mediates host response to cutaneous infection by regulating neutrophil recruitment and adhesion receptor expression

**DOI:** 10.1371/journal.pone.0310810

**Published:** 2024-11-07

**Authors:** Katie D. Pool, Gracie J. Hemmat, Robert A. Dorschner

**Affiliations:** 1 Department of Dermatology, University of California San Diego, San Diego, CA, United States of America; 2 Division of Trauma, Surgical Critical Care and Burn, Department of Surgery, University of California San Diego, San Diego, CA, United States of America; Arizona State University, UNITED STATES OF AMERICA

## Abstract

Rapid neutrophil recruitment is critical for controlling infection, with dysfunctional neutrophil responses in diseases like diabetes associated with greater morbidity and mortality. We have shown that the leukocyte protein ECRG4 enhances early neutrophil recruitment to cutaneous wounds and hypothesized that ECRG4 regulates the early host response to infection. Using a cutaneous infection model, we found that ECRG4 KO mice had decreased early neutrophil recruitment with persistent larger lesions, increased bacterial proliferation and systemic dissemination. Although previous work identified ECRG4 as a negative regulator of CD44 on neutrophils, the mechanism regulating neutrophil recruitment remained unknown. We demonstrated that pro-inflammatory responses were intact in ECRG4 KO mice, but found decreased neutrophil mobilization from bone marrow and decreased migration to chemokines. ECRG4 KO mouse neutrophils demonstrated an increase in adhesion molecules that regulate recruitment, including enhanced induction of integrin CD11b and increased L-selectin and CD44 on bone marrow neutrophils. Analysis of gene expression in leukocytes from diabetic patients found decreased ECRG4 expression with similar increased L-selectin and CD44. We propose a previously unrecognized mechanism governing neutrophil recruitment, whereby ECRG4 mediates neutrophil surface adhesion molecules that determine both recruitment and outside-in signaling that modulates neutrophil response to pro-inflammatory stimuli.

## Introduction

A rapid host response to cutaneous infection is essential to contain and eradicate pathogens, preventing systemic dissemination, sepsis and death [1]. The initial host response is driven by innate immunity, including locally released factors that mediate recruitment of innate immune cells, such as neutrophils, which are the first leukocytes to arrive, deploying an arsenal of antimicrobial mediators. Local production of cytokines, such as IL1β, IL6 and TNFα help amplify this proinflammatory response, while potent chemokines guide the neutrophils to the infection [2–5]. Diseases characterized by neutropenia, such as myelodysplastic syndrome or cytotoxic chemotherapy treatment, or diseases with impaired neutrophil recruitment and function, such as type-2 diabetes mellitus (T2DM), are marked by the risk of more severe infections with increased morbidity and mortality [2, 3, 6, 7].

Neutrophil recruitment to the site of injury or infection is a tightly regulated multi-step process [2]. Neutrophils are maintained in bone marrow (BM) reserves and marginated pools through a balance of adhesion molecules, chemokines and their receptors [8, 9]. In response to an insult, they are rapidly mobilized into the circulation, where adhesion molecules mediate binding to inflamed vasculature near sites of infection and extravasation into tissues. Neutrophils home to the site of infection through recognition of chemoattractant gradients, including intermediate-target chemoattractants, like the chemokine CXCL2, and end-target chemoattractants produced at the site of infection, such as complement product C5a.

Neutrophil surface adhesion molecules, including selectins and integrins, coordinate neutrophil recruitment and function by both directing adhesion to the vasculature and regulating signaling pathways that control neutrophil function. L-selectin is the primary neutrophil selectin and promotes the initial capture of neutrophils by the vasculature to initiate rolling, but is subsequently proteolytically shed after integrin binding to enhance migration and extravasation [10]. It has also been shown to regulate outside-in signaling by the neutrophil β_2_-family integrin macrophage antigen-1 (Mac-1) [11], which is comprised of the common β subunit CD18 and the α_M_ subunit CD11b, and is one of the most important integrins for neutrophil recruitment [10]. The Mac-1 complex, and CD11b itself, bind to ligands on the vasculature to direct rolling and transmigration [2]. Excess CD11b on the neutrophil surface in patients with myelodysplastic syndrome can lead to a relative neutropenia and impaired neutrophil recruitment through excessive binding to the vasculature [6]. This delayed neutrophil recruitment is also seen in animal models via activation of CD11b by small molecule agonists, such as leukadherins [12], or via genetic manipulation to enhance surface CD11b expression [6]. In addition to directly mediating adhesion, these molecules also regulate neutrophil responses though outside-in signaling. Ligand binding by neutrophil adhesion molecules, including CD11b, L-selectin and CD44, can decrease neutrophil responsiveness to key pro-inflammatory signals, such as Toll-like receptor (TLR) signaling [11, 13–18], and regulate neutrophil function, including degranulation, phagocytosis and oxidative burst [19]. Thus, these molecules are essential mediators of the neutrophil response.

We have previously demonstrated that the leukocyte surface protein Esophageal Cancer Related Gene 4 (ECRG4) is important for the early neutrophil response to cutaneous wounds [20]. Loss of ECRG4 expression resulted in delayed neutrophil recruitment to aseptic injuries, which was most remarkable at 24 hours, a time point that is also essential for containing bacterial infection to prevent dissemination. Given the critical importance of a rapid neutrophil response to cutaneous infection, and the severe consequences of impaired neutrophil response seen in patients with neutropenia or T2DM, we sought to evaluate ECRG4 regulated neutrophil recruitment in host defense against cutaneous infection.

In this study, we utilized a cutaneous infection model with the community acquired Methicillin Resistant Staphylococcus Aureus (MRSA) strain, USA300 LAC, as MRSA has become the predominant cause of skin and surgical site infections seen in the ambulatory setting [4, 21]. ECRG4 KO mice developed worse infections with higher local bacterial burden and increased dissemination, which was associated with decreased early neutrophil recruitment. While the local production of proinflammatory signals was intact, ECRG4 KO mice showed decreased neutrophil mobilization from the BM, with decreased migration to major chemoattractants and increased expression of the adhesion molecules L-selectin, CD44 and integrin CD11b. Evaluation of gene expression in leukocytes from diabetic patients demonstrated decreased expression of ECRG4 with a similar pattern of increased adhesion molecule expression that was demonstrated in the ECRG4 KO mouse. This study demonstrates the importance of ECRG4 in regulating the early neutrophil response to cutaneous infection by mediating neutrophil recruitment, and identifies its potential role in the dysfunctional neutrophil response seen in diabetic patients.

## Materials and methods

### Mice

ECRG4 knockout mice on the C57/BL6 background were generated as previously described [20]. 10–12 week old littermate mice were used for experiments involving ECRG4 KOs, and 10–12 week old C57/BL6 purchased from Jackson Laboratory were used as WT mice in experiments without ECRG4 KO mice. Mice were maintained in a specific pathogen free unit on a 12 hr light/12 hr dark cycle with lights off at 6:00 pm and no twilight period. The ambient temperature was 70° ± 1°F and the humidity was 55 ± 5%. Mice were housed using a stocking density of 3–5 mice per cage in individually ventilated caging (Tecniplast GM500) receiving 60 air changes per hour. Aspen chips bedding was provided along with standard environmental enrichment: one cardboard paper towel roll, replaced as needed. Mice were given water and diet *ad libitum*. All mice are given Teklad Traditional Rodent Diet by Intotiv (Cat# 7912). For all experiments, mice were housed individually and for the infection model animals were housed in an ABSL-2 facility with a 3-day acclimation period; all other parameters were maintained. All animal use in this study was carried out in strict accordance with the recommendations in the Guide for the Care and Use of Laboratory Animals of the National Institutes of Health. The protocol was approved by the Institutional Animal Care and Use Committee of the University of California, San Diego (Protocol Number: S07339). All surgery was performed under isoflurane anesthesia, and all efforts were made to minimize suffering.

### Bacterial culture

The MRSA strain USA300 LAC from our previous work [20] and USA300 LAC::*Lux* was a generous gift from Dr. Alexander Horswill [22, 23]. Culture conditions were based on established methods for these bacteria [22–24]. Briefly, MRSA strains were grown overnight at 37°C on Baird-Parker Agar (BPA; VWR Cat#89407–366), with individual colonies of *Staphylococcus* confirmed by their grey/black coloration and coagulase positivity of *S*. *aureus* colonies further identified by a surrounding clear halo; USA300 LAC::*Lux* colonies were also confirmed by their bioluminescence on the plate. Individual colonies were then inoculated in Tryptic soy broth (TSB; Sigma Cat# T8907-500G) at a pH of 7.1–7.5 and grown at 37°C with shaking at 250rpm overnight, before re-inoculation at a ratio of 1:100 and regrown 3 hours to mid-log phase, identified by OD_600_ of 0.8–1.0. Bacteria washed in sterile PBS and resuspended to an OD_600_ of 0.1, corresponding to a concentration of 1x10^8^ CFU/mL. Serial dilutions of the bacteria were prepared in sterile PBS and plated on BPA for enumeration. These plates were incubated overnight at 37°C and colonies counted to confirm concentrations. Counting was performed manually of black colonies with appropriate halos; if non-black colonies lacking halos (and/or bioluminescence for USA300 LAC::*Lux* colonies) were identified, the culture would be considered contaminated and not used.

### Cutaneous infection assay

Cutaneous MRSA infection studies were modified from the protocol of Miller et al [22]. Briefly, mice were anesthetized with 3% isoflurane and dorsal hair was clipped and depilated with Nair® (Church & Dwight). 10^7^ CFU of bacteria in 100μl sterile PBS were injected intradermally using a 30-gauge insulin syringe. For infection timecourse experiments, digital photography was used to image each wound daily with a ruler included for scale. Area was assessed by planimetry using the NIH ImageJ software. Infections were excised at various time points in some experiments. For flow cytometry, lesions or uninvolved skin were digested with a Whole Skin Dissociation Kit (Miltenyi Biotec, #130-101-540) per manufacturer’s instructions to isolate cells. Lesions or uninvolved skin were homogenized with a FastPrep bead homogenizer in Trizol for qPCR or in sterile PBS for bacterial enumeration on TSA plates or ELISA; lung and spleen were also harvested in this way for enumeration of bacterial burden. Blood was harvested by cardiac puncture and RBCs lysed prior to use in flow cytometry or qPCR using Biolegend RBC Lysis Buffer (10X) #420301 diluted in DI Water. BM was harvested from each mouse by excising both tibias and femurs, removing all muscle and cutting off the epiphyses. A hole was punched into a 0.6ml Eppendorf tube with a 16G needle and nested into a 1.7ml Eppendorf tube, and prepared bones placed into the 0.6ml tube. After centrifuging at room temperature for 30” at 16,060xg, the BM pellet in the 1.7ml tube was resuspended in sterile PBS, filtered through a 70μm cell strainer (Fisher Scientific Cat# 22-363-548) and immediately used in analyses. All experiments were repeated 3 times, unless otherwise stated, with data pooled and total N noted in the figure legends of each experiment.

### Systemic lipopolysaccharide (LPS) challenge assay

Mice were anesthetized with 3% isoflurane, weighed and injected intraperitoneally with 10 mg/kg LPS (Sigma, cat# L4130) in sterile saline or equal volume sterile saline control using a 27-gauge needle. After 2 hours, mice were anesthetized again for terminal cardiac puncture to collect blood and harvest of BM (as above).

### Flow cytometry

Live cells were collected from mouse BM, blood or digests of mouse infection and skin, as described above, and flow cytometry performed as previously described [20]. Cells were incubated with a cocktail of primary antibodies that included these specified antibodies at a dilution of 1:50 for 10 minutes at 4°C in the dark (all per manufacturer’s recommendations): anti-CD45-VioGreen (Miltenyi Biotec, 130-110-803), anti-Ly6G-VioBlue (Miltenyi Biotec, 130-119-986), anti-CD11b-APC-Vio770 (Miltenyi Biotec, 130-109-288), anti-CD44-APC (Miltenyi Biotec, 130-119-121) and anti-CD62L-PE-Vio770 (Miltenyi Biotec, 130-112-838). Propidium iodide (Miltenyi Biotec, 130-093-233) exclusion was used to determine viable cells. Flow cytometry and data analysis was performed on a Miltenyi Biotec MacsQuant10 Flow Cytometer, as previously described [20], with supplemental figure S1 Fig demonstrating the gating strategy used. Briefly, the flow cytometer was run on its medium flow speed and neutrophils were identified as CD45^+^ singlet cells that were Ly6G^+^ and CD11b^+^ (see S1 Fig). Data analyzed with MacsQuant(Miltenyi Biotec) and FlowJo software (FlowJo LLC) and statistical analysis performed as below. All experiments were repeated 3 times, unless otherwise stated.

### Migration assay

3μm pore 12mm Transwell inserts (Corning 3415) were coated with 7.5μg/cm^2^ of fibronectin (Calbiochem Cat# 341635-1MG) in sterile water for 2 hours at 37°C, then allowed to dry overnight (Corning protocol CLS-AN-150). Mouse BM was harvested as above, cells counted with a hemocytometer and adjusted to 2x10^6^cells/ml in 0.5% FCS DMEM (Life Technologies Cat# 12430062). 200μl of cell suspension was added to the transwell and 600μl of 0.5% FCS DMEM with indicated chemoattractant was added to the bottom well in a 24 well plate. Cells were incubated for 3 hours at 37°C and 5% CO_2_, then 60μl of 0.5M EDTA added to the bottom of the well and incubated for 15 min at 4°C. Migrated cells in the bottom wells were collected for analysis via flow cytometry.

### qRT-PCR

RNA was isolated from tissue using Zymo Direct-zol RNA Miniprep Plus (Zymo #R2072) according to manufacturers recommended procedures and yields and purity were assessed using a NanoDrop2000 Spectrophotometer (Thermo Fisher). cDNA synthesis was accomplished with 1 μg total RNA using iScript (BioRad #170–8891) according to manufacturer’s procedures. qPCR was performed on a BioRad CFX96 thermocycler with automated threshold settings, standard cycling parameters (95°Cx3min followed by 40 cycles of 95°Cx10sec, 55°Cx10sec, 72°Cx30sec) and utilization of a melt curve analysis to ensure specific product amplification. Primers were purchased from Qiagen, including the housekeeping gene GAPDH, and used at the manufacturer’s recommended final dilution of 1:10. Relative gene expression was analyzed using the delta-delta Ct method, with GAPDH as the housekeeping gene.

Qiagen Primer PCR Cat # 249900 and Geneglobe IDs: IL1b- QT01048355; IL1-R1- QT00095256; IL1-Rn- QT00096054; IL-6- QT00098875; TNFa- QT00104006; CXCL1- QT00115647; CXCL2- QT00113253; CXCL12- QT00161112; CXCR2- QT00283696; CXCR4- QT00249305; CSF3R- QT00150675; FPR1- QT00258139; C5aR1- QT00288232

### ELISA

ELISAs were done using the R&D Systems ELISA kits (TNFa # DY410, G-CSF # DY414, CXCL12 # MCX120, CXCL1 # DY453, CXCL2 # DY452, IL6 # M6000B, IL1b # MLB00C) according to manufacturers recommended procedures. In the case of the DuoSet kits plates and reagents were prepared using the R&D Systems’ DuoSet ELISA Ancillary Reagent Kit 2 (R&D Systems # DY008). Absorbance of the plates was measured using the BMG Labtech FLUOstar Omega Plate Reader using the wavelength specified in the R&D Systems’ ELISA protocol.

### Statistical analysis

All statistical analyses were performed using Graphpad Prism version 10.2.0 (Graphpad Prism Software, Inc). Unpaired two-sample t-test, 2-way ANOVA with Bonferroni post-test, and one-way ANOVA with Sidak’s multiple comparisons test, were used. A P value <0.05 was considered statistically significant. Data are presented as means ± standard deviation of the mean. Sample size calculations performed with alpha 0.05 and beta 0.2.

## Results

### ECRG4 deficient mice develop more severe cutaneous infections with increased systemic dissemination

**Fig 1:**
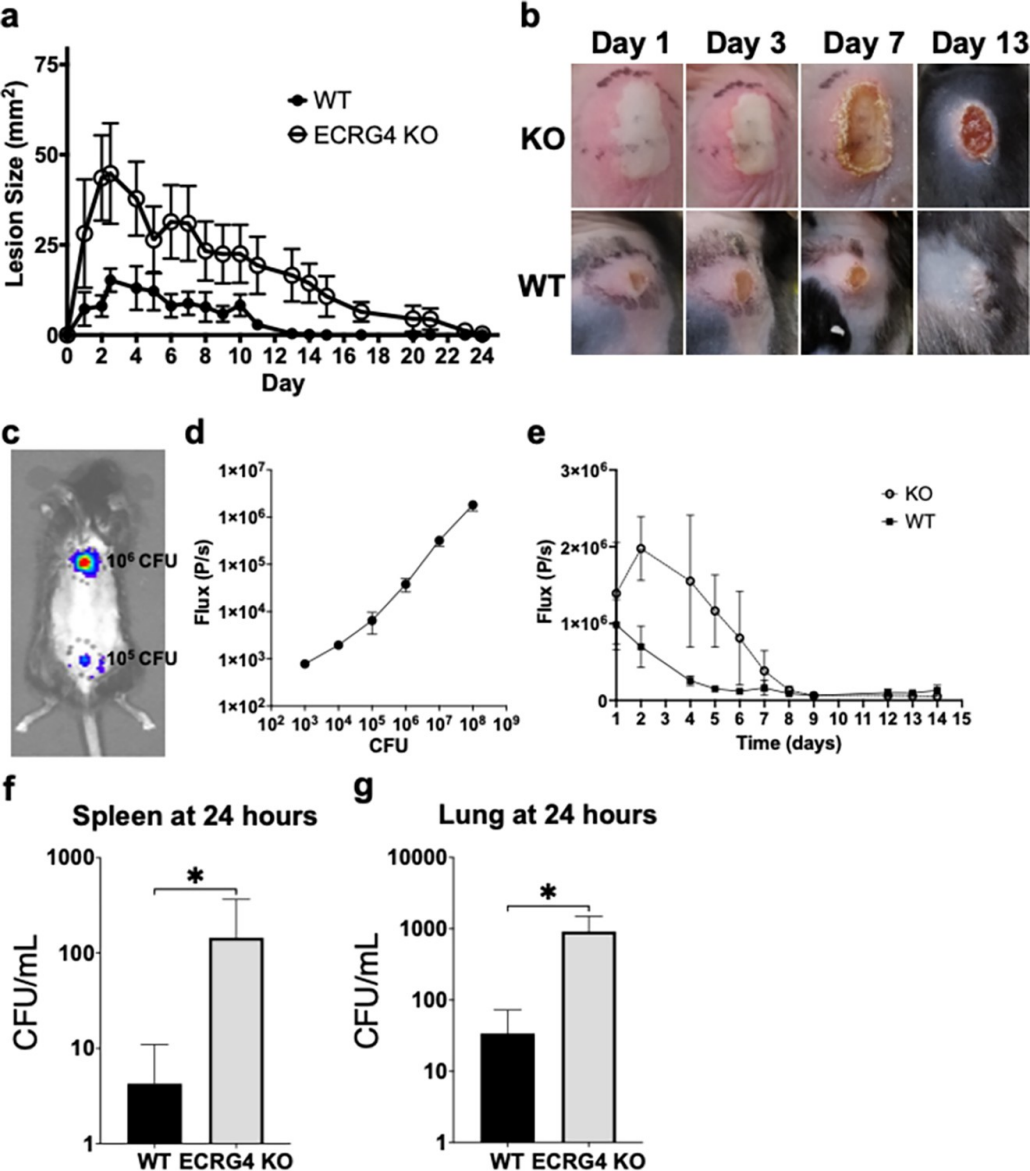
Loss of ECRG4 results in more severe cutaneous MRSA infections with systemic dissemination of bacteria. (a) Time course tracking lesion size in ECRG4 KO (◯) and WT (λ) littermates in response to cutaneous MRSA infection (P<0.001 by 2 way ANOVA and N = 9 mice per group), with representative images (b). (c) Representative image from the IVIS system showing detection of two different inocula of MRSA USA300 LAC:*Lux* (10^6^ CFU cranial and 10^5^ CFU caudal) and plot (d) demonstrating correlation of recovered CFU with measured flux. (e) Time course of MRSA bacterial burden in the cutaneous MRSA infection: WT (ν) and ECRG4 KO (◯) mice, n = 7 and P = 0.014 2-Way ANOVA. Measurement of bacteria in spleen (f, n = 9 and P = 0.048) and lung (g, n = 5 and P = 0.042) at 24 hours after MRSA cutaneous infection; two tailed unpaired t-test.

Neutrophil recruitment is an essential component of the early response to infection, limiting the ability of pathogens to proliferate and spread. Previous work had demonstrated a role for ECRG4 in the early recruitment of neutrophils to cutaneous wounds, leading us to hypothesize that ECRG4 may regulate this critical response to infection. Using an intradermal MRSA infection model with the community acquired strain USA300 LAC, we evaluated the response of ECRG4 KO mice to cutaneous infection. Time course experiments comparing ECRG4 KO mice to wild type (WT) controls demonstrated that ECRG4 KO mice rapidly developed much larger lesions after infection. We found 4-fold larger lesions in the ECRG4 KO mouse by 24 hours (WT 7.22 mm^2^ vs KO 28.13 mm^2^), which increased to a maximal difference of 5-fold at day 2 (WT 8.05 mm^2^ vs KO 43.55 mm^2^), and a peak in overall lesion size at day 3 (WT 15.21 mm^2^ vs KO 44.78 mm^2^) (Quantified in Fig 1A, with representative images in Fig 1B). These larger lesions persisted longer, with WT mice resolving their lesions by day 13 and ECRG4 KO mice persisting until day 24 (Fig 1A and 1B).

Next, we utilized a bioluminescent MRSA USA300 LAC:*Lux* [23] to quantify bacterial burden within the lesion in vivo. We used an in vivo imaging system to non-invasively measure flux from the cutaneous infection, which we determined correlated with CFU of live bacteria in vivo (Fig 1C example imaging of 2 different in vivo CFU inocula and Fig 1D shows correlation). Following cutaneous infection with USA300 LAC:*Lux*, we found that bacteria rapidly proliferated in the ECRG4 KO infection, with flux peaking at day 2, while the WT mouse was able to limit early proliferation, demonstrating declining burden from day 1 (Fig 1E). Bacterial burden in the ECRG4 KO mice began to decrease after day 2 and was reduced to undetectable levels by day 8, whereas the WT mice had undetectable MRSA by day 5. Given the dramatic increase in bacterial burden early in the infection in ECRG4 KO mice, we evaluated mice for systemic dissemination from the cutaneous infection site. Enumeration of bacteria in the spleen and lung at 24 hours after infection demonstrated increased MRSA in the ECRG4 KO mouse, indicating that these mice were unable to contain the cutaneous infection, resulting in systemic spread (Fig 1F and 1G).

### Loss of ECRG4 expression impairs early neutrophil recruitment to infection

**Fig 2:**
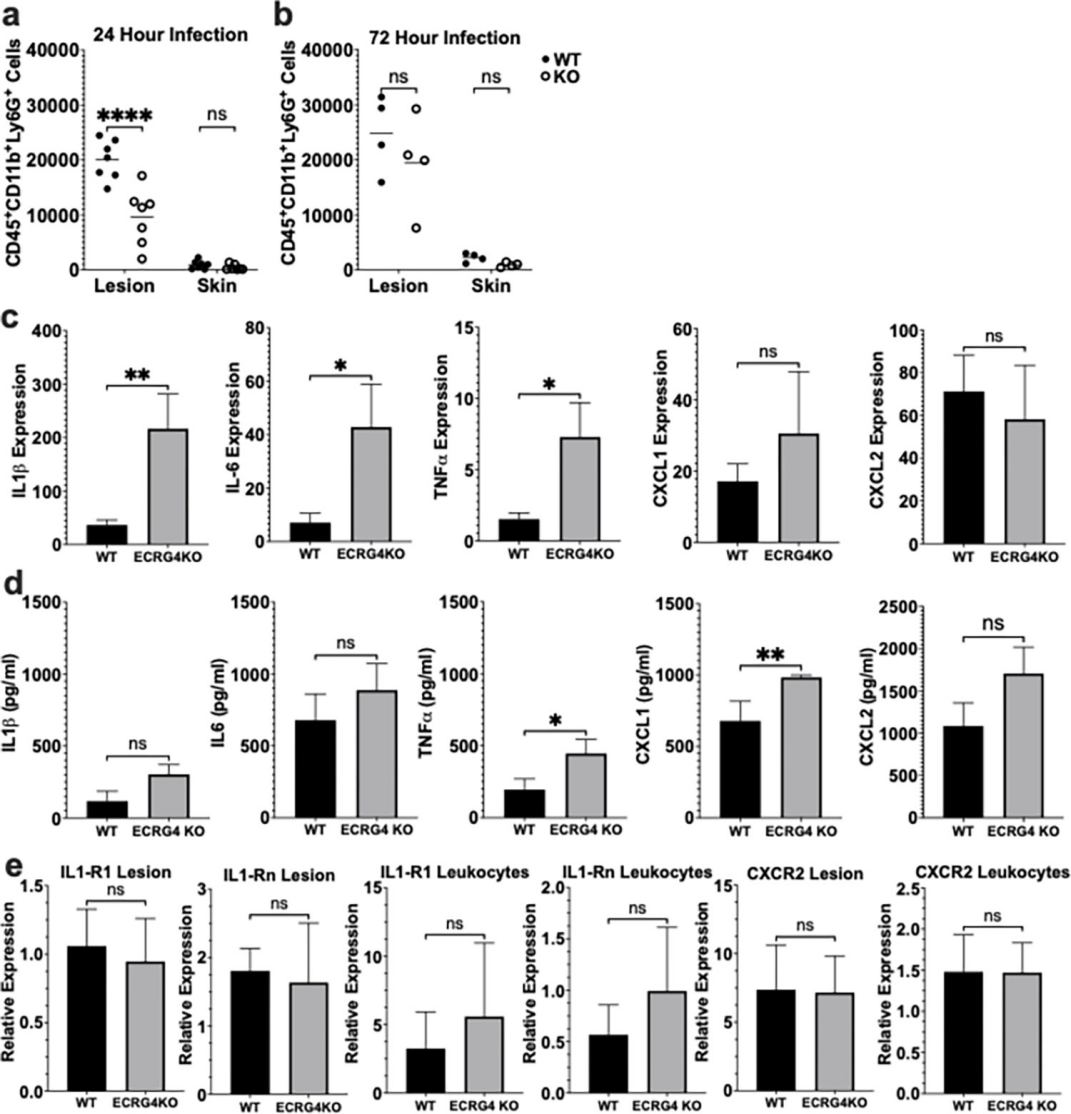
Loss of ECRG4 impairs early neutrophil recruitment but not the local cutaneous cytokine response to infection. Enumeration of CD45^+^CD11b^+^Ly6G^+^ neutrophils recruited to the site of cutaneous infection with MRSA at 24 (a) and 72 (b) hours via flow cytometry. Evaluation of key neutrophil recruitment cytokines and chemokines within the cutaneous infection at 24 hours was performed via qPCR for relative expression of genes (c) and ELISA for expression of protein (d). Expression of receptors for IL-1β and CXCL2, as well as the IL1-R1 antagonist, IL1-Rn, was determined within the lesion and on circulating leukocytes at 24 hours after infection by qPCR (e). N = 7 mice per group. a and b 2-Way ANOVA, c-e unpaired t-test; P = *<0.05, **<0.01, ***<0.001, ****<0.0001.

The initial response to cutaneous injury and infection involves rapid recruitment of neutrophils to control pathogens, which is mediated through local production of proinflammatory cytokines and chemoattractants. Given the importance of this neutrophil response, we evaluated whether the ECRG4 KO mouse had deficient neutrophil recruitment that might be responsible for the observed worse infection and bacterial dissemination (Fig 1). Using flow cytometry to immunophenotype [20] cells (S1 Fig) at the site of infection, we observed that the ECRG4 KO mice had a 50% decrease in neutrophil recruitment at 24 hours (P<0.0001) (Fig 2A). This deficit in neutrophil recruitment relative to the WT control mice narrowed by 72 hours (Fig 2B), demonstrating a trend of 20% fewer neutrophils in the ECRG4 KO infection. This defective early neutrophil recruitment correlates with our finding of a peak difference in lesion size and bacterial burden by day 2 (Fig 1A–1E), with the ECRG4 KO mouse demonstrating decreasing lesion size and bacterial burden by day 4 (Fig 1A and 1E).

### ECRG4 KO mice retain a robust local pro-inflammatory response to infection

In order to evaluate why the ECRG4 KO mice were unable to mount an effective early response to infection, we examined the inflammatory milieu of the cutaneous infection at 24 hours. Production of proinflammatory cytokines and chemokines at the site of infection are important mediators of neutrophil recruitment. Using qPCR, we evaluated the expression of cytokines that are important for neutrophil recruitment to sites of infection, including IL1β, IL-6 and TNFα, as well as the neutrophil chemokines CXCL1 and CXCL2. Given the decreased number of neutrophils recruited to the site of infection, we hypothesized that there may be a decreased production of these proinflammatory mediators in the ECRG4 KO infection. On the contrary, we found that the ECRG4 KO mouse infection had increased expression of IL1β (P = 0.0053), IL-6 (P = 0.047) and TNFα (P = 0.024), with a trend towards increased CXCL1 (Fig 2C). Next, we used ELISA to compare the transcriptional findings with protein at the site of infection. Similar to the findings in our transcriptional analysis, we found increased TNFα (P = 0.028) and CXCL1 (P = 0.0056) in the ECRG4 KO mouse infection, as well as a trend towards increased CXCL2, IL1β, and IL6 (Fig 2D). These data demonstrated that the deficient early recruitment of neutrophils to the site of infection in ECRG4 KO mice was not due to impaired local cytokine and chemokine production. Instead, the ECRG4 KO lesions demonstrated higher levels of these pro-inflammatory mediators, possibly a compensatory response to the insufficient neutrophil recruitment (Fig 2A) and higher bacterial burden (Fig 1A–1E).

Having demonstrated deficient neutrophil recruitment in the ECRG4 KO infection despite intact local proinflammatory mediator production, we next used qPCR to evaluate the expression of genes that regulate neutrophil responses to the cytokine IL-1β and chemokines CXCL1 and CXCL2, as these are critical to early neutrophil recruitment and abscess formation in MRSA infection [25, 26]. There was no change in the expression of the receptors IL1-R1 and CXCR2 within the site of infection or on the circulating leukocytes (Fig 2E). Similarly, expression of the IL1-R1 antagonist, IL1-Rn, which regulates IL1-R1 signaling, was not altered in the ECRG4 KO mouse lesions or circulating leukocytes (Fig 2E). These findings demonstrated that while ECRG4 KO mice have a deficient early neutrophil recruitment to cutaneous infection, they have intact proinflammatory cytokines and chemokines responses.

### Mobilization of neutrophils from bone marrow reserves is decreased in ECRG4 KO mice

**Fig 3:**
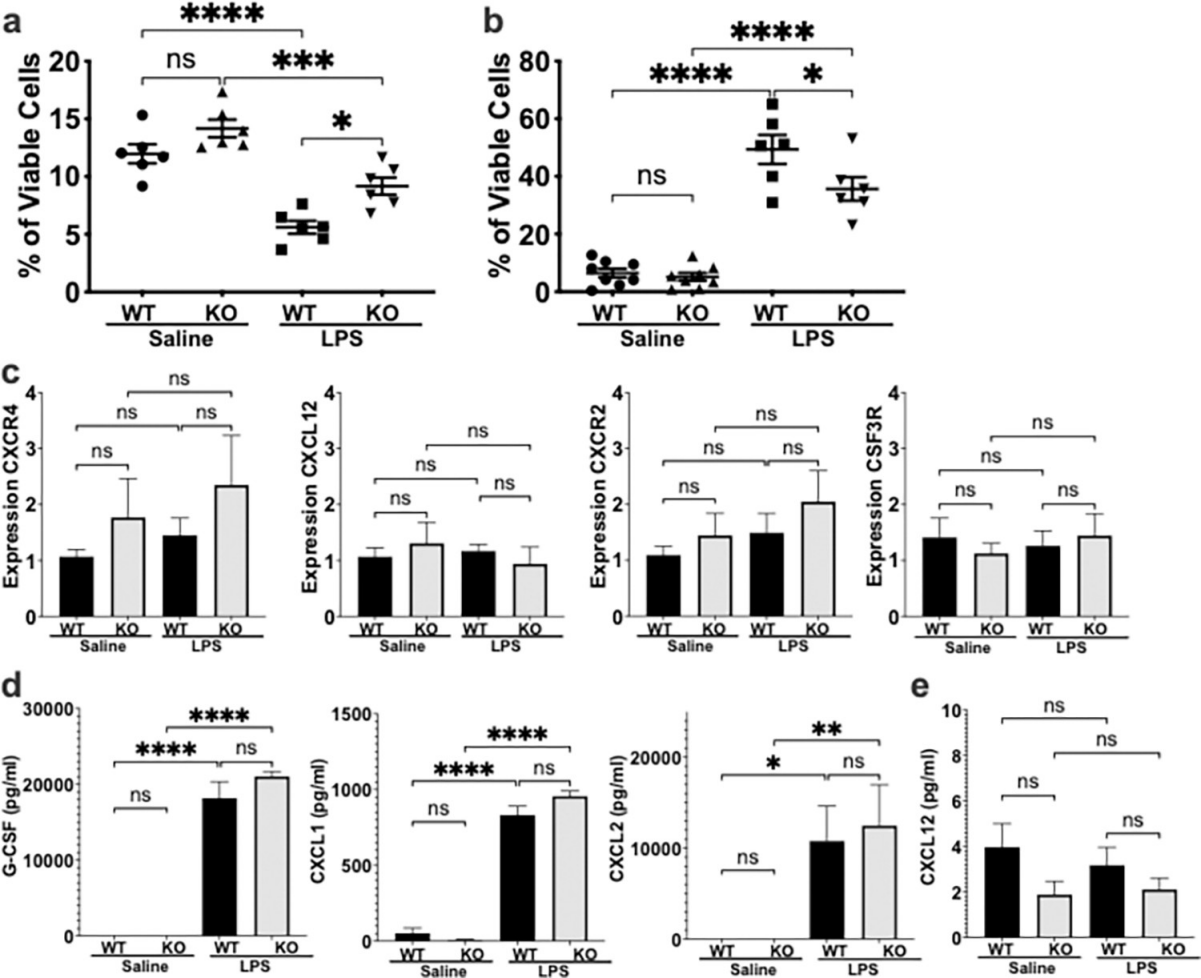
Loss of ECRG4 expression decreases neutrophil mobilization from bone marrow reserves. Early neutrophil mobilization from the BM was determined using an intraperitoneal LPS injection model, with evaluation at 2 hours after challenge. Flow cytometry was used to quantify neutrophils in the BM (a) and blood (b) 2 hours after LPS challenge. The relative expression of key mediators of neutrophil retention in the BM, CXCR4 and CXCL12, as well as the receptors for the major chemokines/cytokines responsible for their mobilization, CXCR2 and G-CSF receptor (*csf3r*) were assessed in the BM by qPCR (c). (d) ELISA was used to evaluate plasma levels of key cytokines and chemokines 2 hours after LPS challenge, and to determine CXCL12 in the BM (e). N = 6 mice per group. 1-Way ANOVA, P = *<0.05 **<0.01, ***<0.001, ****<0.0001.

In the mouse, the BM reserves of mature neutrophils are the primary source of neutrophils mobilized in response to infection or other challenges [8]. We evaluated BM mobilization in the ECRG4 KO mouse using a systemic LPS challenge model. Two hours after intraperitoneal (IP) challenge with LPS or saline control, we utilized flow cytometry to assess neutrophil mobilization out of BM and into the circulation. In saline control mice, there was no difference in the number of neutrophils in the BM of WT vs ECRG4 KO mice (Fig 3A). In response to LPS challenge, we found that ECRG4 KO mice mobilized significantly fewer neutrophils from their BM reserves, as compared to WT mice (P = 0.01, Fig 3A). While WT mice mobilized 60% of their neutrophils at this early time point (WT neutrophils decreased from 11.97% to 5.55% of BM cells, P<0.0001), ECRG4 KO mice only demonstrated a 35% decrease (KO neutrophils decreased from 14.17% to 9.17% of BM cells, P<0.001) (Fig 3A). This correlated with a decrease in the number of neutrophils mobilized into the blood of ECRG4 KO mice, as compared to WT controls (Fig 3B). There was a 7.65-fold increase in circulating neutrophils in the WT mice (WT neutrophils increased from 6.45% to 49.36% of circulating leukocytes, P<0.0001), as compared to 5.9-fold increase in the ECRG4 KO mouse (KO neutrophils increased from 5.99% to 35.66% of circulating leukocytes, P<0.0001) in response to LPS challenge (Fig 3B). These data demonstrate that ECRG4 is important for regulating neutrophil mobilization from the BM into circulation, a critical initial step for the recruitment of neutrophils to the site of infection.

Maintenance of the BM neutrophil reserve is dependent on a balance of chemokines and receptors. Expression of CXCR4 and CXCL12 within the BM promotes neutrophil retention, while certain cytokines in the circulation promote mobilization out of the BM into the blood. To determine if ECRG4 mediates the mobilization of neutrophils from BM via regulation of this system, we evaluated the expression of CXCR4 and CXCL12 in the BM of ECRG4 KO and WT mice. In both saline and LPS-treated mice, we found that there was no difference in the expression of BM CXCR4 or CXCL12 in the ECRG4 KO mice as compared to WT controls (Fig 3C). There was a trend towards increased CXCR4 in the ECRG4 KO mouse, which could promote retention of neutrophils in the BM, however this did not reach statistical significance. Additional evaluation of its binding partner CXCL12 by ELISA confirmed there was no difference in CXCL12 in the BM (Fig 3E). Next, we evaluated G-CSF, CXCL1 and CXCL2 in circulation, as these are key cytokines regulating the mobilization of neutrophils out of the BM in response to injury or infection. First, we used qPCR to determine that the ECRG4 KO mouse BM neutrophils had intact expression of CXCR2 and CSF3R (Fig 3C), the receptors for CXCL1/2 and G-CSF, respectively. ELISA was performed to evaluate circulating cytokines in plasma 2 hours after IP LPS challenge. The ECRG4 KO mouse demonstrated no difference in circulating levels of G-CSF, CXCL1 or CXCL2 in response to systemic challenge; both WT and ECRG4 KO mice showed robust increases in plasma levels in response to the LPS challenge (Fig 3D). These findings are consistent with our earlier results demonstrating that the ECRG4 KO infection has normal local proinflammatory cytokine production (Fig 2). Together, these data demonstrate decreased early mobilization of neutrophils from the BM reserve in the ECRG4 KO mouse, with no differences in chemokine or receptor expression in the BM or production of relevant inflammatory cytokines and chemokines in circulation. This supported a hypothesis that ECRG4 regulates neutrophil mobilization via mechanisms distinct from the production of pro-inflammatory cytokines or alteration of their receptors.

### The responsiveness of neutrophils to chemoattractants is regulated by ECRG4

**Fig 4:**
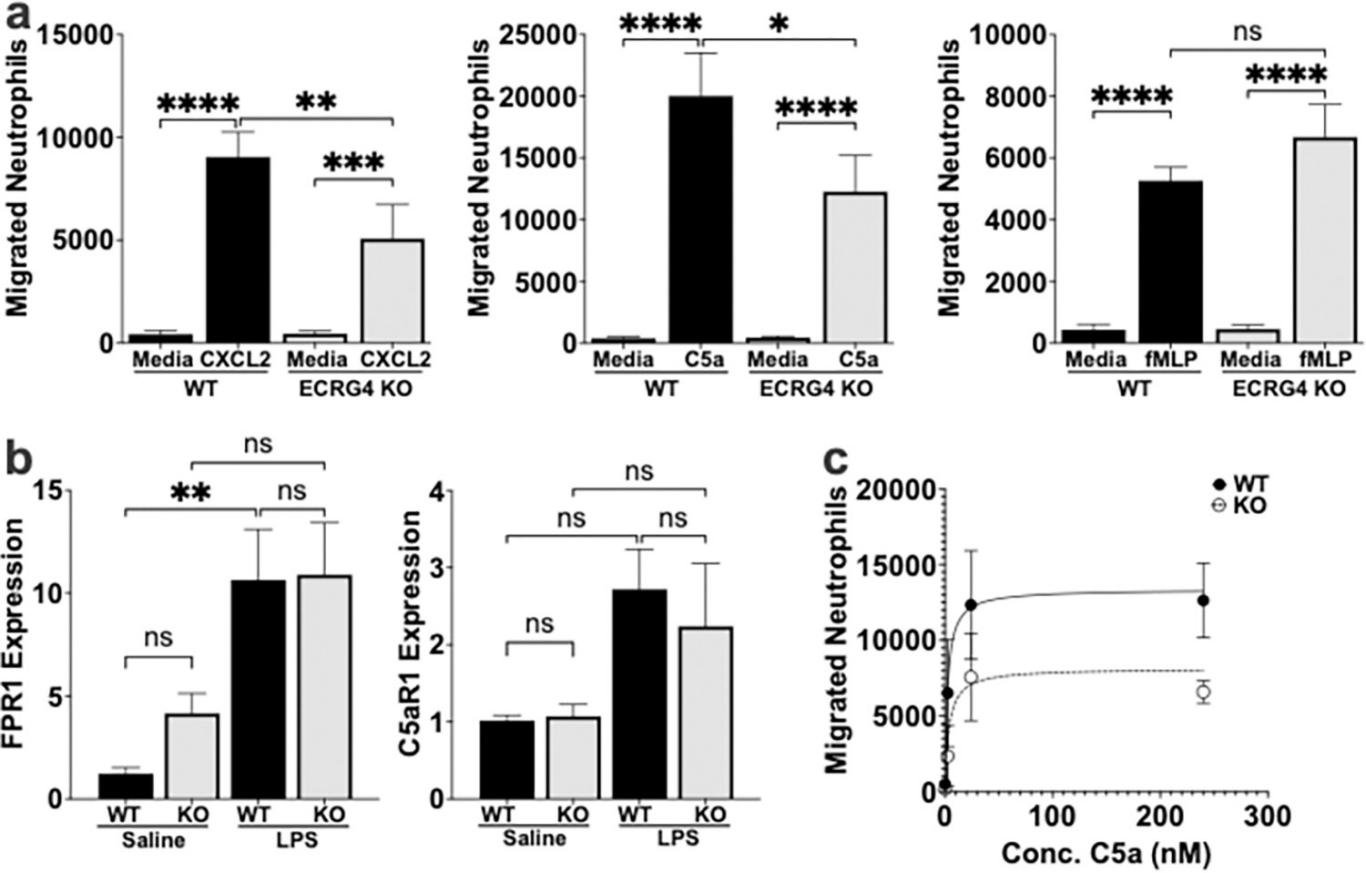
ECRG4 regulates neutrophils migration to chemoattractants. Migration of neutrophils from ECRG4 KO and WT mice to well-defined chemoattractants was assessed in transwell migration assays. Neutrophil migration to 24nM CXCL2, 10nM C5a or 100uM fMLP was determined in fibrinogen coated transwell migration systems, using flow cytometry to determine neutrophil migration at 2 hours (a). Expression of the fMLP receptor, FPR1, and the C5a receptor, C5aR1, was evaluated by qPCR for both control and LPS challenged leukocytes (b). A dose curve of neutrophil migration to C5a was performed (c). N = 10. 1-Way ANOVA, P = *<0.05 **<0.01, ***<0.001, ****<0.0001.

Since our data indicated that there was not a difference in the production of pro-inflammatory mediators by the ECRG4 KO mouse, we evaluated the ability of these neutrophils to migrate to classic neutrophil chemoattractants as a potential mechanism for the neutrophil recruitment deficit seen in the ECRG4 KO infection. Recruitment of neutrophils relies on their ability to sense and migrate towards chemoattractants, including the initial mobilization towards intermediate-target chemoattractants, like the chemokine CXCL2, and then end-target chemoattractants, like C5a and fMLP, to home to the site of infection. We employed transwell migration assays to evaluate the ability of these chemokines to induce ECRG4 KO mouse neutrophil migration (Fig 4A). Despite the fact that there was no difference in CXCL2 production or expression of its receptor, CXCR2 (Figs 2 and 3), we found that ECRG4 KO neutrophils had 56% decreased migration to CXCL2 on fibronectin-coated transwells (9,050 WT neutrophils vs 5,082 KO neutrophils, P = 0.0036). We also found that ECRG4 KO neutrophils had 60% decrease in migration to the end-target chemokine C5a (20,025 WT neutrophils vs 12,277 KO neutrophils, P = 0.0011) (Fig 4A), which occurred over a range of concentrations (Fig 4C). There was no difference in recruitment to the end-target bacterial product, fMLP, in these experiments. Evaluation of the expression of their receptors by qPCR identified no difference in the fMLP receptor, FPR1, or the C5a receptor, C5aR1 (Fig 4B), consistent with the absence of changes in CXCR2 expression (Figs 2E and 3C). These data demonstrate that the ECRG4 KO neutrophils have a decreased capacity to migrate to both an intermediate target and end target chemokine. Since these chemokines signal through distinct receptors and pathways, these data suggested that impaired migration of ECRG4 KO neutrophils may be due to a shared step, such as neutrophil surface adhesion molecules.

### ECRG4 regulates neutrophil adhesion molecule expression

Based on the ability of ECRG4 to regulate neutrophil recruitment and migration that was independent of the production of chemokines, cytokines or their receptors, we evaluated the ability of ECRG4 to regulate adhesion molecules on the neutrophil. Expression of cell surface adhesion molecules, such as integrins and selectins, mediate neutrophil adhesion and rolling on endothelium, extravasation and overall recruitment [2, 8, 25]. The integrin CD11b and L-selectin are well characterized neutrophil adhesion molecules involved in these processes, as well as having been implicated in outside-in signaling, BM retention and mobilization [8, 10, 19]. Our previous work had also shown increased CD44 expression on ECRG4 KO neutrophils during their response to cutaneous wounding [20]. CD44, which canonically binds hyaluronic acid, is another adhesion molecule involved in neutrophil recruitment [27–29], as well as playing an important role in decreasing neutrophil pro-inflammatory responses [15, 16, 30]. We used the systemic LPS challenge model to evaluate the neutrophil surface expression of CD11b, L-selectin and CD44 in vivo via flow cytometry.

**Fig 5:**
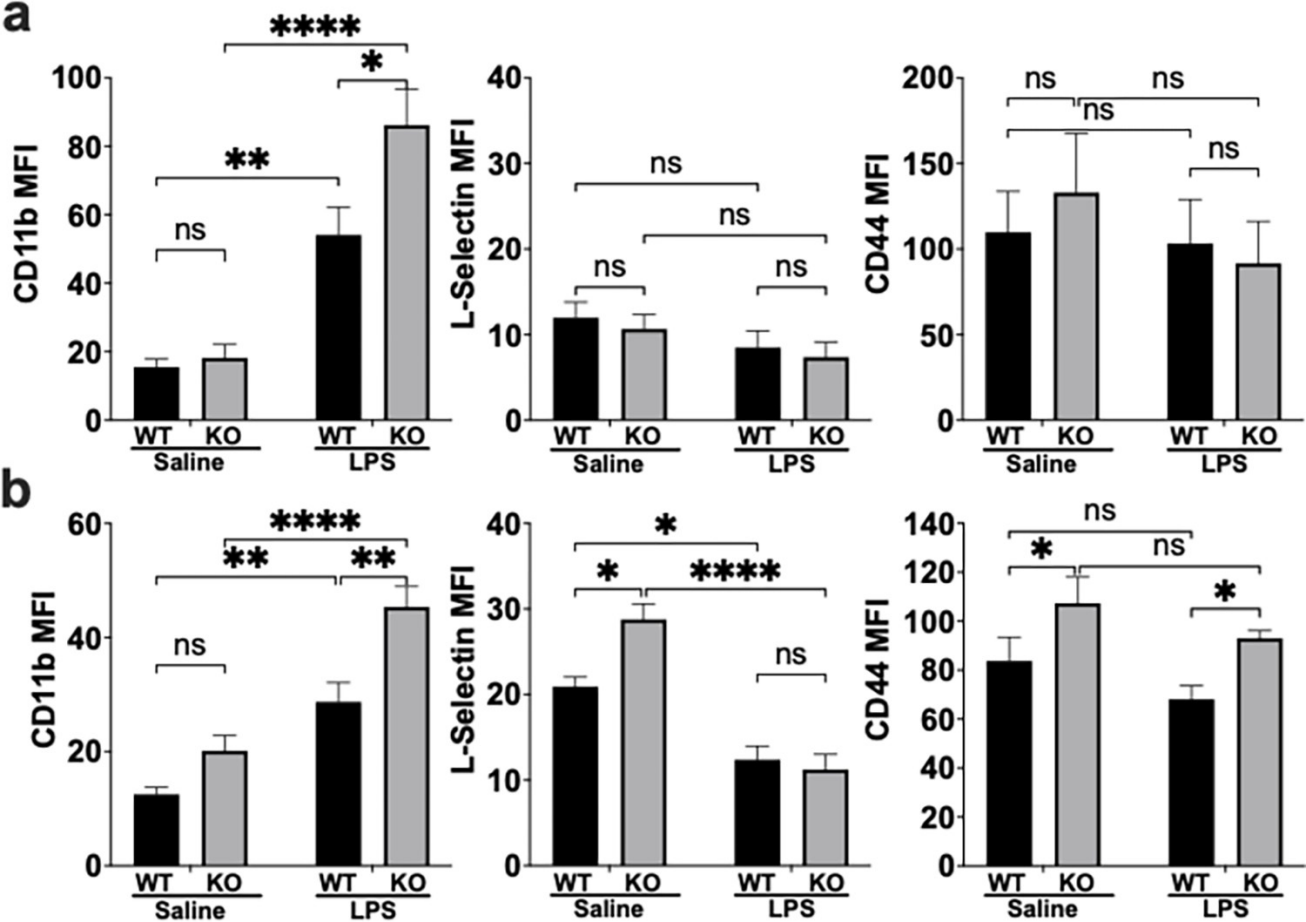
ECRG4 regulates neutrophil surface expression of adhesion molecules. Neutrophil cell surface expression of the integrin CD11b (left), L-selectin (Middle) and CD44 (right), was evaluated by flow cytometry mean fluorescence intensity (MFI) on circulating neutrophils (a) and neutrophils retained in the BM (b) 2 hours after systemic LPS challenge or sterile saline control. N = 6 mice per group. 1-Way ANOVA, P = *<0.05 **<0.01, ***<0.001, ****<0.0001.

In the saline control mice, we found similar expression of CD11b on the surface of ECRG4 KO and WT mouse neutrophils in both circulation (Fig 5A) and in the BM (Fig 5B). Upon activation, neutrophils are known to increase surface expression of CD11b. This was seen after our LPS challenge in both the circulating neutrophils and those retained in the BM. However, after LPS challenge, the ECRG4 KO neutrophils had almost 60% more surface CD11b expression that the WT mouse on circulating neutrophils (MFI on WT 54.13 vs KO 86.18, P = 0.022; Fig 5A) and neutrophils retained in the BM (MFI on WT 28.72 vs KO 45.37, P = 0.0014; Fig 5B).

L-selectin is another key adhesion molecule on the neutrophil surface that is shed upon activation to facilitate rolling and mediate integrin signaling, as well as having been implicated as a BM retention factor [8]. Evaluation by flow cytometry demonstrated the expected decrease in surface L-selectin on both WT and KO circulating neutrophils after the LPS challenge, with about 35% shedding of surface L-selectin for both (Fig 5A). In the BM, however, we found that the ECRG4 KO mouse had 37.5% more surface L-selectin on unchallenged neutrophils (MFI on WT 20.92 vs KO 28.78, P = 0.027), but underwent similar shedding after LPS challenge (P<0.0001) (Fig 5B), suggesting another factor contributing to increased ECRG4 KO BM neutrophil retention.

Finally, we identified increased CD44 protein on ECRG4 KO mouse BM neutrophils, consistent with our previous wound models [20]. ECRG4 KO BM neutrophils had 22% more CD44 (MFI on WT 83.7 vs KO 107.35, P = 0.046) in the saline control mice, and 26.8% more after LPS challenge (MFI on WT 68.1 vs KO 93.1, P = 0.036) (Fig 5B). These experiments demonstrate that ECRG4 can regulate the expression of key neutrophil surface adhesion receptors responsible for mediating neutrophil mobilization from the BM, tethering, and rolling along the endothelium and ultimate migration to the site of injury or infection.

### Diabetic patient leukocytes have decreased ECRG4 and increased adhesion molecule expression

**Fig 6:**
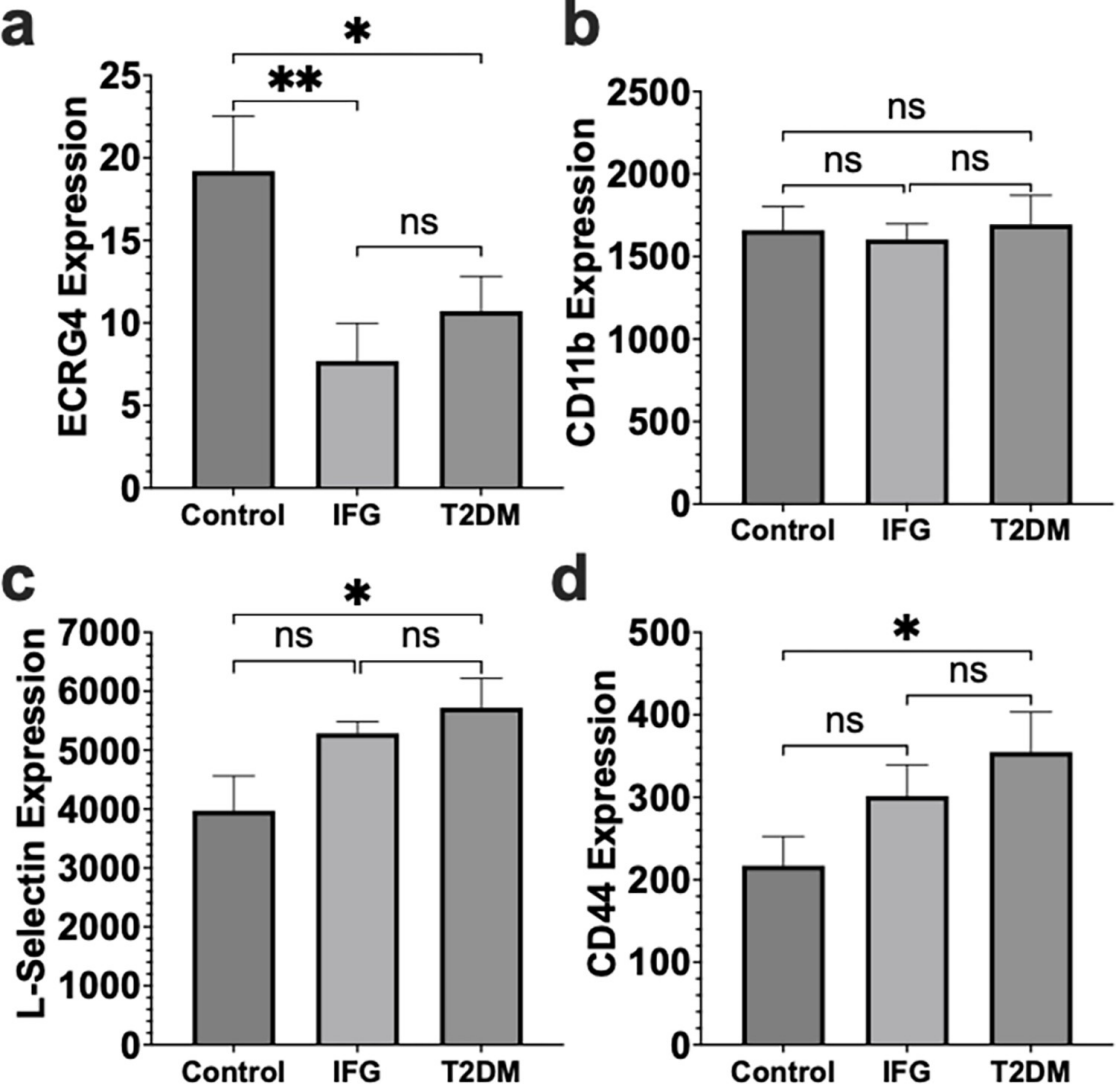
ECRG4 and adhesion molecule expression in diabetic patients. Gene expression in circulating leukocytes from patients with type 2 diabetes (T2DM) (N = 9), impaired fasting glucose (IFG) (N = 7) and healthy volunteers (control) (N = 8) was analyzed from an Illumina beadchip assay [Gene Expression Omnibus accession GSE26168 [36]] (Illumina HumanRef-8 v3.0 expression beadchip). Gene expression for ECRG4 (a), CD11b (b), L-selectin (c) and CD44 (d). 1-Way ANOVA, P = *<0.05 **<0.01.

Patients with diabetes have a well-known defect in neutrophil function and recruitment [7, 31], contributing to their increased risk of infection and development of chronic wounds [31–33]. Additionally, clinical studies have reported increased CD11b and L-selectin on leukocytes from diabetic patients with serious sequelae [34, 35]. Given the importance of ECRG4 in regulating early neutrophil recruitment in wound healing [20] and infection in mice, we hypothesized that it also regulates neutrophil recruitment in humans and evaluated its expression in patients with T2DM. Gene expression data from circulating leukocytes of T2DM patients, patients with impaired fasting glucose (IFG) and healthy controls (Controls) was analyzed from a previously published Illumina HumanRef-8 v3.0 Gene Expression BeadChip experiment (GEO GSE26168) [36] using the NCBI GEO2R analysis tool. These samples were from adult (21–70 years old) male patients who were not on any medications and were stratified based on WHO definitions into Controls (fasting glucose <6.1mmol/L), IFG (fasting glucose from 6.1–7.0 mmol/L) and T2DM (fasting glucose ≥ 7.0 mmol/L). Expression of ECRG4 in circulating leukocytes from IFG and T2DM patients was 60% (P = 0.0065) and 44% (P = 0.042) less than the level detected in healthy controls, respectively (Fig 6A). In the uninjured ECRG4 KO mice, we observed similar amounts of CD11b on the neutrophil, but increased L-selectin and CD44 (Fig 5A and 5B saline controls). We observed a similar pattern in these human leukocytes from otherwise healthy subjects, with no difference in the expression of CD11b in the control vs IFG and T2DM patients. Compared to the controls, however, the T2DM leukocytes demonstrated 31% increased expression of L-selectin (P = 0.015) and 61% increased CD44 (P = 0.032), with an increasing trend in both L-selectin and CD44 in the IFG patients (Fig 6B–6D). These results demonstrate that patients with T2DM, as well as IFG, have decreased expression of leukocyte ECRG4, with increased adhesion molecule expression, similar to our observations in the ECRG4 KO mouse.

## Discussion

This work demonstrates for the first time that ECRG4 is an important regulator of the host defense against cutaneous infection and identifies decreased ECRG4 expression in diabetic patient leukocytes as a potential contributor to the neutrophilic dysfunction in these patients. In our mouse model, loss of ECRG4 expression impairs the early recruitment of neutrophils to cutaneous infection, resulting in more severe infection and bacterial proliferation. The local proinflammatory response is not impaired by the loss of ECRG4, but migration of neutrophils to potent chemoattractants CXCL2 and C5a is reduced and mobilization of neutrophils from the BM reserves is decreased. We identify an increase in expression of the adhesion molecule CD11b (α_M_ integrin) on activated neutrophils from the ECRG4 KO mouse, as well as increased expression of L-selectin and CD44 on neutrophils in the BM. Previous studies have determined that increased CD11b expression and activation can impair neutrophil recruitment [6, 12, 18, 37, 38], thus providing a potential mechanism for the observed delay in ECRG4 KO mouse neutrophil response to infection. Evaluation of human leukocytes demonstrated that both IFG and T2DM patients have decreased ECRG4 expression with increased adhesion molecule expression, mirroring our observations in the ECRG4 KO mouse. These findings are significant, as diseases with dysfunctional neutrophil recruitment, including diabetes, are marked by an increased risk of severe infection and mortality [2, 39]. Our study identifies a previously unrecognized mechanism regulating early neutrophil recruitment.

Disease states that interfere with neutrophil recruitment result in more severe infection [2–4, 6, 7, 25, 31, 39–44]. Here, we find that ECRG4 KO mice have deficient neutrophil recruitment to cutaneous infection with MRSA, with more severe infection and dissemination of bacteria. Despite this phenotype, we did not find any deficits in the production of pro-inflammatory cytokines or chemokines, or deficiencies in their receptors. Mobilization from the BM is a key step in neutrophil recruitment. In mice, this is the primary source of mature neutrophils [8, 45]. This BM reserve of neutrophils is maintained via a balance of cytokines and receptors, with the interaction between CXCR4 and CXCL12 playing a central role in maintaining the neutrophils in the BM, while G-CSF, CXCL1 and CXCL2 stimulate mobilization into circulation [45]. We found that there was a decrease in the mobilization of neutrophils from the ECRG4 KO BM in response to a systemic LPS challenge. This was not due to deficient plasma chemokine production or deficient expression of their receptors on the BM neutrophils. Similarly, this was not due to an imbalance in the CXCR4/CXCL12 retention system within the BM. Therefore, we sought alternative mechanisms regulating the ability of neutrophils to emigrate from the marrow.

Appropriate recruitment of neutrophils to the site of infection requires neutrophils to follow various chemoattractant gradients. Initially, intermediate-target chemoattractants, such as the chemokine CXCL2, guide the neutrophils in circulation to intravascular sites near the source of infection, where they can then bind to the activated vascular endothelium and extravasate into the tissue. Next, potent end-target chemoattractants take over, with neutrophils preferentially following these signals, such as C5a, to their final target [25]. This process relies on both receptors for the chemokines, but also adhesion molecules regulating neutrophil binding to the vascular endothelium, rolling and signaling cytoskeletal rearrangement that allow extravasation and crawling through the tissue. In our study, we found decreased neutrophil migration to potent chemoattractants, including CXCL2 and C5a. These chemokines signal via distinct pathways and evaluation of their receptors demonstrated no difference in expression, suggesting that ECRG4 may be regulating other components responsible for neutrophil migration that are shared by both intermediate-target and end-target chemokines.

This defect in migration to chemokines and BM mobilization, in combination with the preserved pro-inflammatory cytokine and chemokine production, led us to investigate the expression of cell surface adhesion molecules, which are key regulators of neutrophil recruitment [2, 8, 25, 39]. We identified altered expression of the neutrophil surface adhesion molecules CD11b and L-selectin in the ECRG4 KO mice, both of which regulate the rate of neutrophil mobilization and recruitment to the site of infection [2, 5, 6, 8, 10–12, 37, 38]. CD11b is an abundant integrin that forms a heterodimer with CD18 on neutrophils to form the Mac-1 complex. CD11b is increased on the surface of activated neutrophils, and plays a central role in neutrophil rolling, extravasation and function. Indeed, therapeutic strategies to inhibit neutrophil recruitment in inflammatory and autoimmune disease by blocking CD11b has been beneficial in some experimental models, but has not been as successful as a therapeutic in human clinical trials [12]. Interestingly, enhancing CD11b binding also led to decreased neutrophil recruitment with improved outcomes in several disease models by retarding neutrophil recruitment [12, 18]. These findings correlate with a study by Mei *et al*. that demonstrated increased neutrophil CD11b in patients with myelodysplastic syndrome del(5q), which results in relative neutropenia, due to adhesion and extravasation of neutrophils, and an increased risk of infection [6]. When Mai modeled this disease with a *mDai1* deficient mouse, they confirmed that increased surface CD11b, as a result of reduced endocytosis, impaired neutrophil migration and caused the relative neutropenia. Together, these studies support our finding that ECRG4 may mediate neutrophil recruitment via its regulation of CD11b, with a loss of ECRG4 leading to increased CD11b on the surface and subsequent delayed recruitment. Given the role of endocytosis in regulating neutrophil surface adhesion molecules, it is interesting to speculate that ECRG4 may function by modulating adhesion receptor endocytosis, as ECRG4 has been shown, in vitro, to associate with other receptors, such as CD14, to enhance endocytosis [46, 47]. This is an area of ongoing investigation. Similarly, we identified increased expression of CD44 on the ECRG4 KO mouse neutrophil, similar to findings in the response to cutaneous wounds. CD44 is abundant on neutrophils and its expression has also been shown to increase neutrophil adhesion [27–30]. Finally, L-selectin (CD62L) is important for regulating the rate of neutrophil rolling and has been suggested to be a BM retention factor [10, 11, 45]. L-selectin is known to regulate the velocity of neutrophil rolling, with shedding of surface L-selectin increasing neutrophil recruitment [2, 10]. Our studies did not identify a difference in L-selectin on circulating neutrophil, suggesting that regulation of its shedding may not be part of ECRG4’s mechanism. However, we did identify increased L-selectin on the surface of BM neutrophils, potentially enhancing BM retention and positioning it as another mechanism for reduced neutrophil mobilization from the BM of the ECRG4 KO mouse.

In addition to the direct role of adhesion in regulating neutrophil recruitment, these molecules have been shown to regulate the neutrophil inflammatory response through a process of outside-in signaling [13, 17–19, 48]. For instance, ligand binding of CD11b can lead to decreased responsiveness to TLR signaling, in part through the degradation of the key adaptor proteins TRIF and MyD88 [13, 17]. CD11b activates Syk to phosphorylate MyD88 and TRIF, with subsequent degradation of these adaptor proteins by Cbl-b and resultant impaired TLR signaling. Recently, L-selectin was also shown to be an important regulator of integrin outside-in signaling [11], with the shed L-selectin enhancing integrin signaling by increasing Mac-1 clustering on the neutrophil surface, promoting effector function. Additionally, numerous reports have demonstrated the anti-inflammatory role of CD44 signaling, particularly in decreasing neutrophil response to TLR signals and reducing neutrophil recruitment [15, 16, 28, 30, 49], including via direct interaction to block the intracellular TIR domain and indirectly by enhancing A20 expression. Therefore, the increase in neutrophil surface CD11b, CD44 and L-selectin on ECRG4 KO neutrophils can directly restrain neutrophil recruitment through increased adhesion, as well as decrease neutrophil response to PAMPs/DAMPs via reduced TLR signaling, thus providing a mechanism for the delayed neutrophil recruitment with more severe infection seen in the ECRG4 KO mouse.

The epidemic of T2DM, a chronic disease marked by neutrophil dysfunction with increased morbidity from infection and chronic wounds, is a major strain on our health care systems. As part of this neutrophil dysfunction, prior studies observed altered expression of adhesion molecules, including CD11b and L-selectin, on leukocytes from diabetic patients with complications such as retinopathy, nephropathy and atherosclerosis [34, 35], but did not identify the mechanism. In this study, we identify decreased ECRG4 expression in diabetic patients’ leukocytes with concomitant alteration of adhesion molecule expression mirroring the ECRG4 KO mouse findings, supporting the regulation of neutrophil adhesion molecules by ECRG4 as a mechanism for the neutrophil dysfunction and increased risk of infection in diabetic patients. The role of ECRG4 in regulating inflammation has only recently been identified [20, 47, 50–52] and this is the first description of altered ECRG4 expression in diabetes. The only prior clinical study investigating ECRG4 expression on leukocytes was an evaluation of trauma patients that correlated clinical status with neutrophil ECRG4 expression, but did not investigate a mechanism for this finding [50]. Our analysis of diabetic patient leukocytes is limited to gene expression in otherwise healthy diabetic patients (ie. not infected), but their concordance with our mouse model supports a prospective study in diabetic patients, which has been recently initiated. This will evaluate ECRG4 and adhesion molecule protein expression on the diabetic neutrophil, as well as functional studies to evaluate the importance of this pathway on neutrophil recruitment in this highly prevalent disease. Interestingly, ECRG4 expression has been shown to be regulated by hypermethylation of the promoter, which leads to loss of its expression in various cancers [53, 54]. Recent studies have identified hyperglycemia driven DNA methylation in diabetic patients [55–57], supporting a hypothesis that hypermethylation of the ECRG4 promoter in diabetics may lead to its decreased expression and subsequent overexpression of neutrophil adhesion molecules as a mechanism for impaired neutrophil recruitment in this disease. This will also need to be addressed in our clinical studies and may help identify new therapeutic pathways to address the neutrophil dysfunction, and its sequelae, in this disease.

Some of the most common cutaneous complications of T2DM include the increased risk of infection, with systemic dissemination, and chronic wounds, which together present a significant source of morbidity with high rates of amputation and subsequent mortality [32, 58–60]. Impaired neutrophil response is a cause for the increased risk for infection, and recently impaired early neutrophil recruitment has been identified as a driver for development of chronic diabetic wounds [7, 31, 42, 43]. Recognition of ECRG4 as an important regulator of early neutrophil recruitment, and discovery of its deficiency in T2DM, positions this pathway as a potential therapeutic target, as restoring adequate early neutrophil responses in diabetic injury and infection may prevent more severe infections and correct impaired wound healing. Furthermore, monitoring ECRG4 expression in patients may be useful to stratify patient risk for the development of these devastating sequelae and support either prophylactic strategies for prevention or more aggressive early clinical intervention for this subset of patients who would benefit the most. Indeed, the only prior prospective clinical study evaluating ECRG4 levels on leukocytes was in burn patients, which revealed variation in individual ECRG4 expression that correlated with clinical status [50]. As epigenetic regulation of the ECRG4 promoter regulates its expression in certain cancers [61], this mechanism may also regulate its expression in other physiologic states or diseases, such as T2DM. Understanding this mechanism would not only support the use of ECRG4 expression as a marker for infection and chronic wound risk in T2DM patients, but also provide a target for restoring ECRG4 expression and, thus, restoring neutrophil responses to decrease these risks.

Based on the findings reported here, and prior studies, we propose a mechanism whereby ECRG4 expression regulates neutrophil adhesion molecule expression to modulate the recruitment of neutrophils to sites of injury and infection. This is a novel pathway that is relevant to our understanding of human disease, including the findings that this mechanism may contribute to neutrophil dysfunction in T2DM. Further elucidation of this pathway may enable the use of ECRG4 expression to identify patients at increased risk of diabetic complications, such as severe infections and chronic wounds, as well as identify novel therapeutic targets for preventing these devastating complications.

## Supporting information

S1 Fig Gating strategy for flow cytometry. Representative plots demonstrating the gating strategies used to identify CD45+Ly6G+CD11b+ neutrophils from mice.
(TIF)
