# Supplementary figures and images for: ECRG4 mediates host response to cutaneous infection by regulating neutrophil recruitment and adhesion receptor expression
Source: PLoS One. 2024 Nov 7;19(11):e0310810. doi: 10.1371/journal.pone.0310810 (PMC11542879; doi:10.1371/journal.pone.0310810)

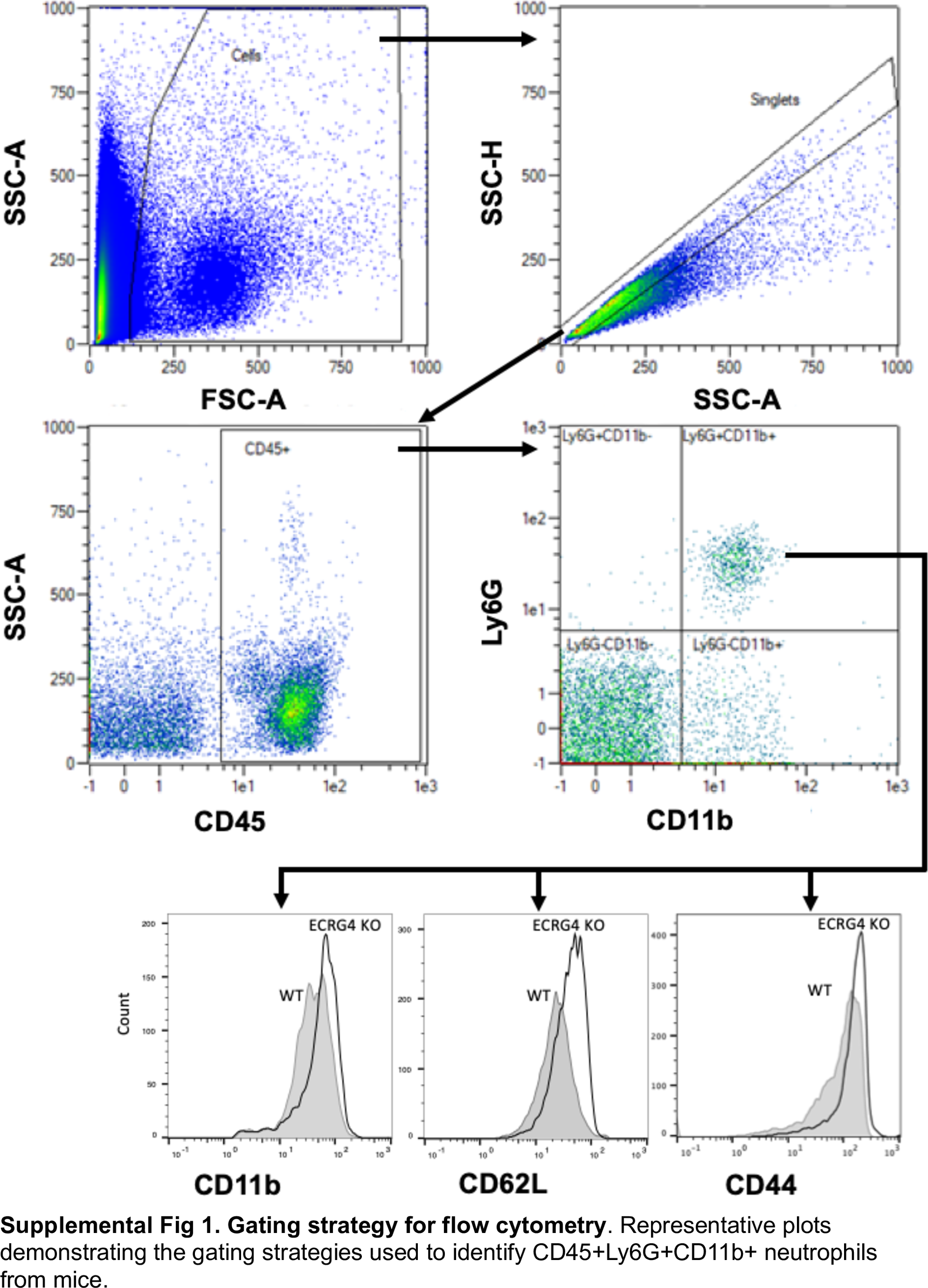

Supplement: S1 Fig — Representative plots demonstrating the gating strategies used to identify CD45+Ly6G+CD11b+ neutrophils from mice. (TIF) [file pone.0310810.s001.tif]
